# Mechanism of TNFα-induced downregulation of salt-inducible kinase 2 in adipocytes

**DOI:** 10.1038/s41598-023-37340-5

**Published:** 2023-06-29

**Authors:** Magdaléna Vaváková, Kaisa Hofwimmer, Jurga Laurencikiene, Olga Göransson

**Affiliations:** 1 Protein Phosphorylation Research Group, Section for Diabetes, Metabolism and Endocrinology, Department of Experimental Medical Science, Lund University, Biomedical Centre C11, Klinikgatan 28, 221 84 Lund, Sweden; 2 Lipid Laboratory, Unit of Endocrinology, Department of Medicine Huddinge, Karolinska Institute, Stockholm, Sweden

**Keywords:** Cytokines, Kinases, Type 2 diabetes, Adipocytes

## Abstract

Salt-inducible kinase 2 (SIK2) is highly expressed in white adipocytes, but downregulated in individuals with obesity and insulin resistance. These conditions are often associated with a low-grade inflammation in adipose tissue. We and others have previously shown that SIK2 is downregulated by tumor necrosis factor α (TNFα), however, involvement of other pro-inflammatory cytokines, or the mechanisms underlying TNFα-induced SIK2 downregulation, remain to be elucidated. In this study we have shown that TNFα downregulates SIK2 protein expression not only in 3T3L1- but also in human in vitro differentiated adipocytes. Furthermore, monocyte chemoattractant protein-1 and interleukin (IL)-1β, but not IL-6, might also contribute to SIK2 downregulation during inflammation. We observed that TNFα-induced SIK2 downregulation occurred also in the presence of pharmacological inhibitors against several kinases involved in inflammation, namely c-Jun N-terminal kinase, mitogen activated protein kinase kinase 1, p38 mitogen activated protein kinase or inhibitor of nuclear factor kappa-B kinase (IKK). However, IKK may be involved in SIK2 regulation as we detected an increase of SIK2 when inhibiting IKK in the absence of TNFα. Increased knowledge about inflammation-induced downregulation of SIK2 could ultimately be used to develop strategies for the reinstalment of SIK2 expression in insulin resistance.

## Introduction

Salt-inducible kinase 2 (SIK2) is a serine/threonine kinase downstream of the master kinase liver kinase B1^1^, with a high expression level in white adipose tissue^2,3^. Previously, we have shown that SIK2 is downregulated in adipose tissue of individuals with obesity and insulin resistance^4^. Pharmacological- or genetic inhibition of SIK isoforms resulted in impairment of insulin signaling and/or glucose uptake in rat and human adipocytes^4,5^. It is thus possible that downregulation of SIK2 in obesity could contribute to the development of obesity-induced insulin resistance.

Obesity and insulin resistance is often associated with a low-grade inflammation in adipose tissue and elevated tissue- and plasma levels of tumor necrosis factor α (TNFα), interleukin (IL)-6, IL-1β and monocyte chemoattractant protein-1 (MCP-1)^6,7^. These pro-inflammatory stimuli signal through a number of intracellular pathways involving serine/threonine kinases such as c-Jun N-terminal kinase (JNK), extracellular signal-regulated kinase (ERK), p38 mitogen activated protein kinase (p38) and inhibitor of nuclear factor kappa-B (NFκB) kinase (IKK)^8,9^. IKK in turn phosphorylates inhibitor of NFκB (IκB), which leads to IκB degradation and subsequent translocation of NFκB to the nucleus^10^.

We^4^ and others^11^ have previously shown that expression of SIK2 mRNA is downregulated by TNFα in human adipocytes and at the mRNA and protein level in 3T3-L1 adipocytes. The mechanism underlying the downregulation of SIK2 by TNFα is not fully understood. Therefore, in this study we investigated involvement of selected key mediators of pro-inflammatory pathways in TNFα-induced SIK2 downregulation in adipocytes. Furthermore, we explored if other cytokines or chemokines have comparable effects on SIK2 to TNFα.

## Results

### SIK2 protein expression is downregulated not only by TNFα but to some extent also by MCP-1 and IL-1β

**Figure 1:**
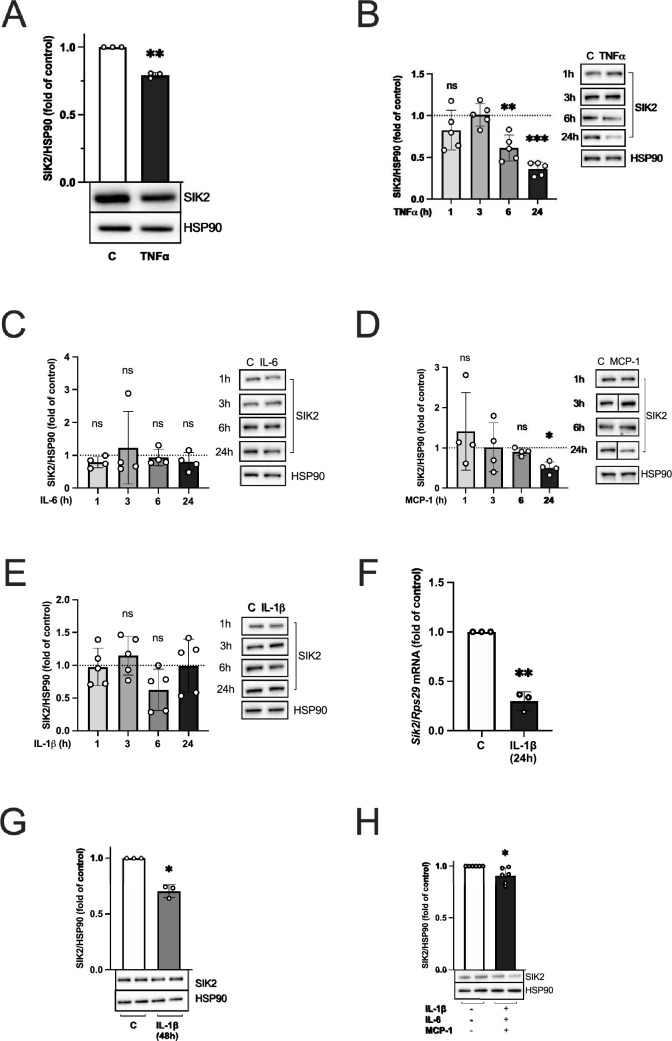
SIK2 protein levels at different timepoints after treatment with TNFα, IL-6, MCP-1, IL-1β and their combination. SIK2 protein levels in hMSCs differentiated into adipocytes (**A**) and 3T3-L1 adipocytes (**B**–**E**, **G**–**H**) treated with or without (control, **C**, dashed line) 50 ng/ml of human TNFα for 24 h (**A**), 20 ng/ml of mouse TNFα (B), 20 ng/ml IL-6 (**C**), 20 ng/ml MCP-1 (**D**) or 20 ng/ml IL-1β (**E**) for indicated time periods. *Sik2* mRNA levels in 3T3-L1 adipocytes treated with 20 ng/ml IL-1β (**F**) for 24 h. SIK2 protein levels after 48 h of 20 ng/ml IL-1β (**G**) or a combination of 100 ng/ml IL-1β, 50 ng/ml IL-6 and 20 ng/ml MCP-1 (**H**) for 24 h in 3T3-L1 adipocytes. HSP90 was used as a loading control for protein analysis. Representative blots are shown. *Rps29* was used as a housekeeping gene for mRNA analysis. Data are presented as means ± SD from multiple independent experiments (**A**, **F**–**G**; n = 3, **C**, **D**; n = 4, **B**, **E**; n = 5, **H**; n = 6). Statistical significance was analyzed by one sample t-test (**A**, **F**–**H**) or by one way ANOVA with a Holm-Šidak’s multiple comparisons test (**B**–**E**). Uncropped blots are presented in Supplementary Fig. S3.

In agreement with our previous study investigating the effect of TNFα on SIK2 mRNA levels^4^, SIK2 protein was downregulated in human mesenchymal stem cells (hMSCs) differentiated into adipocytes and treated with TNFα (Fig. 1A). Successful TNFα stimulation was confirmed by means of increased p38 phosphorylation and decreased IκBα levels (Supplementary Fig. S1A,B). Similarly, in the mouse adipocyte like cell line 3T3-L1, TNFα downregulated SIK2 protein- (Fig. 1B) and mRNA expression (Supplementary Fig. S2A). Efficiency of TNFα treatment was demonstrated by a decrease of IκBα levels (Supplementary Fig. S1C).

To assess if SIK2 responds to inflammatory stimuli other than TNFα, but that are also known to be elevated in obesity^6^, we treated 3T3-L1 adipocytes with IL-6, MCP-1 and IL-1β for indicated time periods (Fig. 1C–G, Supplementary Fig. S1D–F, S2B, C). Again, a decrease in IκBα levels (IL-6 and MCP-1) or an increase in the phosphorylation of p38 (IL-1β), indicated successful cytokine treatment (Supplementary Fig. S1D–F). For IL-6, we did not observe downregulation of SIK2, neither at the protein- (Fig. 1C) nor at the mRNA level (Supplementary Fig. S2B). Notably, we observed significant SIK2 downregulation after 24 h treatment with MCP-1 (Fig. 1D), however without an effect on *Sik2* mRNA at the same time point (Supplementary Fig. S2C). IL-1β did not affect SIK2 protein levels over a 24 h period (Fig. 1E), but significantly reduced *Sik2* mRNA at 24 h (Fig. 1F). To further elucidate this finding, we treated 3T3-L1 adipocytes with IL-1β for 48 h, which, in contrast to the shorter timepoints investigated previously, lead to a 30% downregulation of SIK2 protein levels (Fig. 1G). Finally, to investigate the combined effect of these cytokines on SIK2 protein levels, we co-treated 3T3-L1 adipocytes with IL-1β, IL-6 and MCP-1. This treatment resulted in a 9% decrease in the SIK2 protein level after 24 h (Fig. 1H). Phosphorylation of ERK1/2 and phosphorylation of protein kinase B (PKB) was monitored to determine the effect of the combined cytokine stimulation on inflammatory pathways (Supplementary Fig. S2D,E). Interestingly, the cytokine co-treatment resulted in a decrease of pERK/ERK (Supplementary Fig. S2D). However, we observed a tendency to decreased pPKB/PKB ratio (Supplementary Fig. S2E), which is in line with Yang et al.^12^.

In summary, our results indicate that not only TNFα, but also IL-1β and MCP-1, have negative effects on SIK2 expression. However, IL-1β seems to affect SIK2 protein levels only after extended treatment (48 h) and MCP-1 has no apparent effect on *Sik2* mRNA levels in 3T3-L1 adipocytes. We therefore next focused on investigating involvement of selected key mediators of pro-inflammatory pathways in TNFα-induced SIK2 downregulation.

### TNFα-induced downregulation of SIK2 is not mediated by MEK1, p38, JNK or IKK

To identify pathways involved in TNFα-induced SIK2 downregulation in 3T3-L1 adipocytes, we pharmacologically inhibited kinases known to be activated downstream of the TNFα receptor, namely mitogen activated protein (MAP) kinase kinase 1 (MEK1), p38, JNK and IKK (Fig. 4).

**Figure 2:**
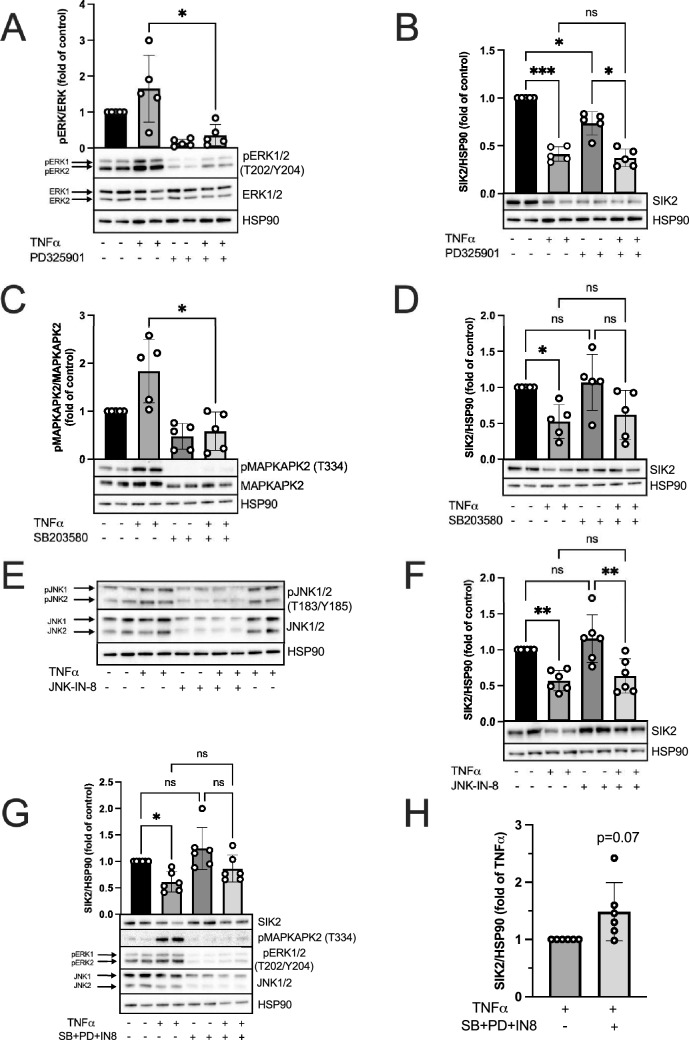
Effect of pharmacological inhibition of MEK1, p38 and JNK on SIK2 protein levels with or without TNFα. Pharmacological inhibition of MEK1 by 1 μM PD325901 (**A**, **B**), p38 by 50 μM SB203580 (**C**, **D**), JNK by 10 μM JNK-IN-8 (**E**, **F**) and the combination of these three inhibitors (**G**, **H**) with or without 20 ng/ml TNFα in 3T3-L1 adipocytes. Cells were incubated with TNFα and their respective inhibitors for 18 h (**A**–**D**, **G**, **H**) or for 19 h (**E**, **F**) which included 1 h of pre-treatment with JNK-IN-8 inhibitor. Efficiency of the inhibitors was monitored by analyzing T202/Y204 pERK1/2/ERK1/2 ratio (**A**, **G**), T334 pMAPKAPK2/MAPKAPK2 ratio (**C**, **G**) and electrophoretic mobility of JNK1/2 (**E**, **G**). SIK2 protein levels were analyzed (**B**, **D**, **F**–**H**) and HSP90 was used as a loading control. Representative blots are shown. Data are presented as means ± SD from multiple independent experiments (**A**–**D**; n = 5, **F**–**H**; n = 6). Statistical significance was analyzed by a two-tailed paired t-test (**A**, **C**), one way ANOVA with a Holm–Šidak’s multiple comparisons test (**B**, **D**, **F**, **G**) or by one sample t-test (**H**). Uncropped blots are presented in Supplementary Fig. S5.

First, MEK1, the upstream kinase for ERK, was inhibited using PD0325901^13^ and the efficiency of the inhibition was validated by monitoring the phosphorylation of ERK1/2 (Fig. 2A). Treatment with PD0325901 resulted in a significantly reduced phosphorylation of ERK1/2 in the presence of TNFα—demonstrating successful inhibition of MEK1. As observed previously, TNFα induced a downregulation of SIK2, which was however not prevented by the MEK1 inhibitor (Fig. 2B). On the contrary, we observed a decrease of SIK2 in response to the inhibitor alone (Fig. 2B).

The pharmacological inhibitor SB203580 was used for inhibiting p38 MAPK and the efficiency was assessed by monitoring phosphorylation of the p38 substrate MAP kinase-activated protein kinase 2 (MAPKAPK2)^14^ (Fig. 2C). When inhibiting p38, the reduction of SIK2 upon TNFα stimulation was no longer significant (Fig. 2D). However, there was also no significant difference in SIK2 levels when comparing TNFα-treated cells in the absence and presence of the inhibitor, although the inhibitor efficiently reduced MAPKAPK2 phosphorylation (Fig. 2C). There was no effect of the inhibitor alone on SIK2 protein levels (Fig. 2D). All in all, our interpretation of these results is that most likely p38 does not mediate the effect of TNFα on SIK2 and the absence of significant TNFα-mediated downregulation in the presence of the SB203580 is due to low sample size.

Next, JNK was inhibited using JNK-IN-8^15^. Efficiency of the inhibitor was verified by a change in the electrophoretic mobility (gel shift) of JNK1/2, with the inhibitor-bound form of JNK migrating higher up, as shown by Zhang et al.^15^ (Fig. 2E). We also observed a reduction in JNK expression over the studied time period (Fig. 2E). However, pre-treatment with JNK-IN-8 did not prevent TNFα-induced downregulation of SIK2 (Fig. 2F). Moreover, JNK-IN-8 alone did not alter SIK2 protein levels (Fig. 2F).

To elucidate a potential compensatory effect of JNK, MEK1 and p38 pathways, when one of them are inhibited, we treated 3T3-L1 adipocytes simultaneously with JNK-IN-8, PD325901 and SB203580 inhibitors. The effect of this mix on pro-inflammatory signaling pathways was assessed by monitoring pMAPKAPK2 and pERK1/2 as well as by gel shift of JNK1/2 (Fig. 2G). Co-inhibition of these pathways tended to partially prevent TNFα-induced downregulation of SIK2, when only comparing cells treated with TNFα in the absence or presence of the inhibitor, however this did not reach statistical significance (Fig. 2H). SIK2 protein levels remained unchanged when treated with beforementioned inhibitors in the absence of TNFα (Fig. 2G).

**Figure 3:**
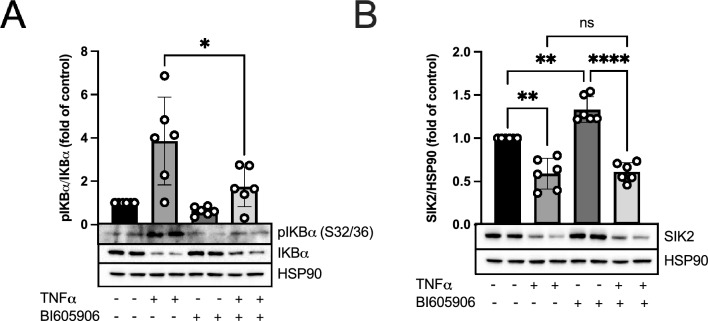
Effect of pharmacological inhibition of IKK on SIK2 protein levels with or without TNFα. Pharmacological inhibition of IKK by 10 μM BI605906 (**A**, **B**) for 18 h with or without 20 ng/ml TNFα in 3T3-L1 adipocytes. Efficiency of BI605906 was monitored by analyzing S32/36 pIκBα/IκBα ratio (**A**). SIK2 protein levels were analyzed (**B**) and HSP90 was used as a loading control. Representative blots are shown. Data are presented as means ± SD from multiple independent experiments (n = 6). Statistical significance was analyzed by a two-tailed paired t-test (**A**) or by a one way ANOVA with Holm–Šidak’s multiple comparisons test (**B**). Uncropped blots are presented in Supplementary Fig. S5.

Finally, to assess the possible involvement of the IKK/NFκB pathway, we treated 3T3-L1 adipocytes with BI605906^16^, an inhibitor of IKK, and validated successful inhibition by monitoring the pIκBα/IκBα ratio (Fig. 3A). Co-treatment of cells with BI605906 and TNFα did not prevent the downregulation of SIK2 by TNFα (Fig. 3B), although a reduction in pIκBα/IκBα indicated successful inhibition (Fig. 3A). However, inhibition of IKK by BI605906 in the absence of TNFα resulted in significant upregulation of SIK2 on the protein level (Fig. 3B).

## Discussion

**Figure 4:**
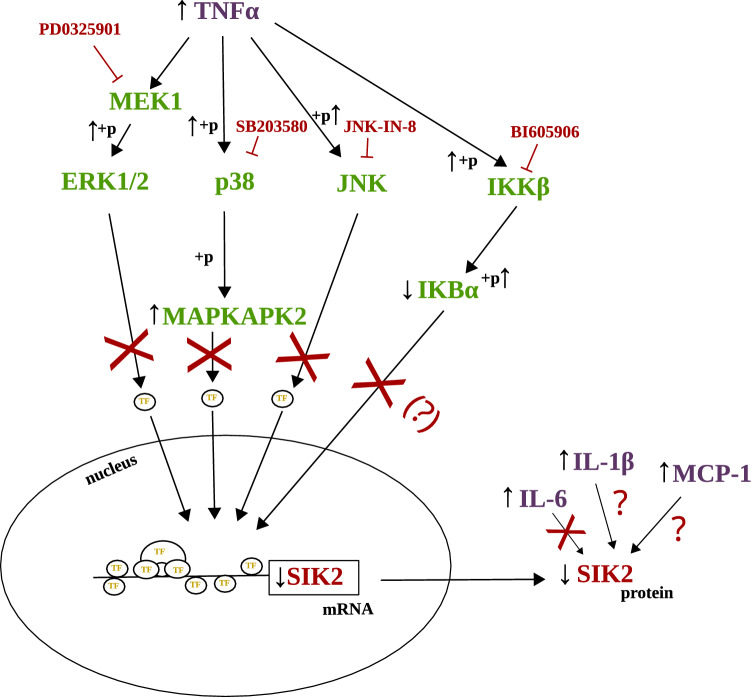
Schematic summary of investigated pathways and main conclusions. We hypothesized that SIK2 is downregulated through one or several highlighted pathways downstream of TNFα, and used a number of pharmacological agents (in red) to inhibit these pathways. We also investigated the potential effects of cytokines other than TNFα, on the expression of SIK2. We conclude that TNFα-induced downregulation of SIK2 is likely not mediated by MEK1, p38, JNK or IKK. However, we do not completely rule out the involvement of IKK. Moreover, SIK2 expression is to some extent downregulated by IL-1β and MCP-1, albeit not as rapidly and robustly as by TNFα. ERK1/2: Extracellular signal-regulated kinase 1/2; IκBα: nuclear factor kappa-B inhibitor alpha; IKKβ: Inhibitor of nuclear factor kappa-B kinase subunit beta; JNK: c-Jun N-terminal kinase 1; MAPKAPK2: mitogen activated protein (MAP) kinase-activated protein kinase 2; MEK1: MAP kinase kinase 1; SIK2: Salt-inducible kinase 2; TF: transcription factor; TNFα: tumor necrosis factor α; IL-6: interleukin 6; IL-1β: interleukin 1 β, MCP-1:monocyte chemoattractant protein-1; + p: phosphorylation; ↓: downregulation; ↑: upregulation; ⊥: inhibition.

We and others have previously demonstrated that SIK2 is downregulated by TNFα on the protein and mRNA level in the 3T3-L1 mouse adipocyte like cell line^4,11^. We have also shown a downregulation of *Sik2* mRNA by TNFα in in vitro differentiated human adipocytes^4^. In this study, we verified these results, and additionally demonstrated that TNFα-induced downregulation of mRNA translates into lower protein levels of SIK2 in the human cells as well. We have also previously shown that SIK2 expression in adipose tissue is negatively correlated with BMI^4^. Obesity is often associated with low grade inflammation and elevated levels of various pro-inflammatory factors, including TNFα, IL-6, IL-1β and MCP-1^6^. In this study we conclude that, in addition to TNFα, IL-1β and MCP-1 have effects on SIK2 expression (Fig. 4), but these are not as rapid and profound as those of TNFα. We therefore hypothesize that inflammation-associated downregulation of SIK2 is mainly driven by TNFα, but that other cytokines also contribute. In future studies it would be interesting to address if low adipose tissue SIK2 expression in obesity and insulin resistance is indeed a result of low-grade inflammation and the presence of elevated TNFα.

MCP-1 downregulated SIK2 protein levels after 24 h, which was however not associated with changes in mRNA at the same timepoint. This may indicate that *Sik2* mRNA levels were downregulated by MCP-1 in a shorter time period and returned to a basal level after 24 h treatment. We can also speculate that MCP-1 affects protein stability and/or degradation of SIK2. Further studies are needed to answer these questions. mRNA analysis revealed that, not only TNFα, but also IL-1β induced *Sik2* mRNA downregulation, but the latter did not translate into lower SIK2 protein until after 48 h treatment. The reasons for this delayed effect on the protein levels are not clear but the long-time frame suggests that the effects of IL-1β might be indirect and secondary to for example IL-1β-induced production of TNFα^17^. Future studies are needed to elucidate this downregulation of *Sik2* by IL-1β in more detail.

An important focus of our work was to delineate mechanisms whereby TNFα downregulates SIK2. Using pharmacological inhibitors, we showed that neither MEK1, JNK nor p38 activity is required for TNFα-induced SIK2 downregulation (Fig. 4). In fact, inhibition of MEK1 in the absence of TNFα resulted in *downregulation* of SIK2 on protein level, suggesting, if anything, that MEK signaling to some extent promotes SIK2 expression. During the preparation of this manuscript, Yoon et al. showed involvement of IKK in TNFα-induced *Sik2* mRNA downregulation, using the inhibitor IKK16^11^, whereas in our experiments, inhibition of IKK with BI605906 did not reverse the reduction in SIK2 expression following TNFα. The reason for this discrepancy is not clear but could involve different time points [18 h (our study) vs. 6 h (Yoon et al.^11^) of co-treatment with TNFα and the inhibitor], different cell models (3T3-L1 vs. primary mouse adipocytes) or different materials analyzed (protein vs mRNA). We attempted to investigate effects of the IKK16 inhibitor on SIK2 protein levels in 3T3-L1 adipocytes, but observed significant cell death at the concentration used by Yoon et al.^11^. Moreover, we did not observe efficient IKK inhibition at lower concentrations (data not shown). Noteworthy, BI605906 is an IKK2-selective inhibitor^16^, whereas IKK16 inhibits both IKK1, IKK2^18^ as well as an unrelated leucine-rich repeat kinase 2 (LRRK2)^19^, suggesting that IKK1 or LRRK2 could be mediating TNFα-induced SIK2 downregulation. Interestingly, we did detect a significant upregulation of SIK2 protein level when inhibiting IKK in the absence of TNFα stimuli. This, together with the data by Yoon et al.^11^, supports involvement of IKK in the regulation of SIK2 expression, at least in the basal state (Fig. 4). Another possibility that could be addressed in future studies is the involvement of non-canonical TNFα signaling, centered around NFκB-inducing kinase (NIK)^20^.

Mechanisms for transcriptional regulation of SIK2, such as the transcription factors involved, have been scarcely studied, but Yoon et al.^11^ demonstrated that overexpression of CCAAT/enhancer-binding protein alpha (C/EBPα) induced SIK2 protein expression as well as the activity of an SIK2 luciferase reporter, proposing that C/EBPα is a direct transcriptional activator of SIK2. Moreover, they showed that overexpression of NFκB, which is activated downstream of IKK^21^, reduced C/EBPα reporter activity. Together, this indicates that the downregulation of SIK2 by TNFα could be mediated via NFκB-induced reduction of C/EBPα. However, considering the rapid effect of TNFα on SIK2 expression, an intriguing possibility is that NFκB directly suppresses SIK2 transcription—a hypothesis that could be tested using SIK2 reporter assays and chromatin immunoprecipitation in future studies.

In summary, we have further established that TNFα downregulates SIK2 expression both at the mRNA- and protein level in mouse as well as in human adipocytes. Furthermore, our results support that other pro-inflammatory cytokines can contribute to regulation of SIK2 expression but that the effects of TNFα are more profound. Neither inhibition of MEK1, p38, JNK nor IKK reversed TNFα-induced SIK2 protein downregulation (Fig. 4). However, IKK inhibition increased SIK2 in the absence of TNFα, suggesting that IKK may indeed be involved in the regulation of SIK2 expression (Fig. 4).

A limitation of this study is the potential off target effects of pharmacological inhibitors. In the future, siRNA silencing could be used to further study the involvement of various kinases in the downregulation of SIK2 by TNFα.

## Methods

### Chemicals and reagents

Mouse TNFα (#T539), human TNFα (#T6674), mouse IL-1β (#SRP8033), mouse IL-6 (#SRP3330) and mouse MCP-1 (#SRP3215) were purchased from Sigma-Aldrich. PD0325901 (#HY-10254), SB203580 (#HY-10256), JNK-IN-8 (#HY-13319), BI605906 (#HY-13019) and IKK16 (#HY-13687) were obtained from MedChemExpress and used in concentrations and time points indicated in figure legends.

### Cell culture

3T3-L1 murine fibroblasts obtained from American Type Culture Collection (ATCC, CL-173) were cultured in high glucose DMEM media (Sigma, #D6429) supplemented with 10% fetal bovine serum (FBS, Sigma, #F7524) and 100 U/ml penicillin/streptomycin (Sigma, #P0781) in 5% CO_2_ humidified atmosphere at 37 °C, at sub-confluence. Two days post-confluent cells were exposed to 1 µM dexamethasone (Sigma-Aldrich #D4902), 0.52 mM 3-isobutyl-1-methyxantine (IBMX, Sigma-Aldrich, #I7018) and 1.74 µM insulin (Sigma-Aldrich, #I2643) for 72 h to induce differentiation. From day three, dexamethasone, IBMX and insulin were removed from the media. Cells were treated as indicated in figures and collected 9–14 days post-differentiation. hMSCs were isolated from a human multipotent stem cell population originating from adipose tissue, cultured and differentiated into adipocytes in vitro as described previously^22^.

### Cell stimulation, lysis and protein extraction

Cells were treated with cytokines and/or inhibitors as indicated in figure legends. Treatment conditions were controlled with an equal volume of H_2_O (TNFα, IL-1β), phosphate buffered saline (PBS) (IL-6, MCP-1) or dimethyl sulfoxide (DMSO) (inhibitors). Cellular proteins were collected in lysis buffer containing 50 mM Tris–HCl pH 7.5, 1% NP40, 1 mM ethylenediaminetetraacetic acid (EDTA), 1 mM ethylene glycol-bis(β-aminoethyl ether)-N,N,N′,N′-tetraacetic acid (EGTA), 5 mM Na-pyrophosphate, 0.27 M sucrose, 50 mM NaF, 1 mM Na-orthovanadate, 1 mM dithiotreitol (DTT) and cOmplete protease inhibitors (Roche, #11697498,001) (3T3-L1) or RIPA buffer (Thermo Scientific, #89901) supplemented with protease inhibitors (Millipore, #539134) and phosphatase inhibitors (Roche, #04906837,001) (hMSCs). Protein concentration was determined by the Bradford method for protein quantification^23^.

### Western blot

Cell lysates (10–30 µg) mixed with 4xLDS sample buffer (Thermo Scientific) were heated for 5 min in 95 °C, loaded on pre-cast Novex Bis–Tris 4–12% polyacrylamide gels (Thermo Scientific), transferred to nitrocellulose membrane (Amersham™ Protran^®^ Western blotting membrane, Sigma-Aldrich), blocked for 60 min in 10% (w/v) skimmed milk in tris-buffered saline containing Tween 20 (TBS-T, 50 mM Tris–HCl pH 7.6, 137 mM NaCl and 0.1% (w/v) Tween 20, Sigma-Aldrich) and incubated overnight at 4 °C in primary antibodies diluted in 5% (w/v) bovine serum albumin (BSA, Sigma-Aldrich) in TBS-T. Horseradish peroxidase-coupled anti-mouse (Sigma-Aldrich, #NA931) and anti-rabbit (Thermo Scientific, #A16096) secondary antibodies, together with enhanced chemiluminescence reagent (SuperSignal West Pico or Femto Chemiluminescent Substrates, Thermo Scientific) were used for developing the signals. Bio-Rad Chemidoc CCD camera and Image Lab software (Bio-rad Laboratories) were used for detection and quantification. Signals were normalized against the loading control (HSP90) and a control condition (cells treated with H_2_O, PBS or DMSO) loaded on the same gel.

Primary antibodies used in this study included anti-HSP90 (BD Biosciences, #BD610418), anti-SIK2 (produced in house by Innovagen, Lund, as described in Henriksson et al.^24^), anti-ERK1/2 [Cell Signaling Technology (CST), #9102], anti-T202/Y204 p-ERK1/2 (CST, #9101), anti-JNK1/2 (Upstate, #06-748), anti-T183/Y185 p-JNK1/2 (CST, #4668), anti-IκBα (CST, #4814), anti-S32/36 p-IκBα (CST, #9246), anti-p38 (CST, #9212), anti-T180/Y182 p-p38 (CST, #9211), anti-MAPKAPK-2 (CST, #12155), anti-T334 p-MAPKAPK-2 (CST, #3007), anti-PKB (CST, #9272) and anti-S473 p-PKB (Thermo Fisher, #44621G).

### RNA extraction and qPCR

Cells were lysed in Qiazol lysis reagent (Qiagen) and RNA was extracted according to the manufacturer’s instructions using the RNeasy Mini kit (Qiagen). One step real time PCR was performed using Quantifast SYBR Green RT-PCR kit (Qiagen) in a QuantStudio 5 Real Time PCR system (Applied Biosystems). The results were calculated using the 2^−ΔΔ^CT method, normalized to the 40S ribosomal protein S29 (*Rps29*) and expressed as fold of a control condition. Primers for *Sik2* (#QT00129269) and *Rps29* (#QT02328858) were obtained from Qiagen.

### Statistics

Data for SIK2 protein levels in samples treated with 20 ng/ml TNFα for 18 h and their representative controls were pooled and tested for normality by Shapiro–Wilk test (n = 28, *p* > 0.1). Normal distribution of the data was thereafter assumed across the study. Data are presented as means ± standard deviation (SD). The statistical significance was analyzed by one-way ANOVA with Holm-Šídák’s multiple comparisons test, two tailed paired t-test or by one sample t-test, as stated in the legends. Statistical analysis as well as visualization was performed using GraphPad Prism 9. *p < 0.05, **p < 0.01, ***p < 0.001, ****p < 0.0001.

## Supplementary Information

Supplementary Information.

## Data Availability

The datasets generated and/or analyzed during the current study are available from the corresponding author on reasonable request.
