# Supplementary material for: Mechanism of TNFα-induced downregulation of salt-inducible kinase 2 in adipocytes
Source: Sci Rep. 2023 Jun 29;13:10559. doi: 10.1038/s41598-023-37340-5 (PMC10310826; doi:10.1038/s41598-023-37340-5)
Supplement: Supplementary file 1 — Supplementary Information. [file 41598_2023_37340_MOESM1_ESM.pdf]

Supplementary information

Mechanism of TNFα-induced down-regulation of salt-inducible kinase 2 in adipocytes

Magdaléna Vaváková[1], Kaisa Hofwimmer[2], Jurga Laurencikiene[2], Olga Göransson[*1]

[1]Protein Phosphorylation Research Group, Section for Diabetes, Metabolism and Endocrinology, Department of Experimental Medical Science, Lund University, Biomedical Centre C11, Klinikgatan 28, 221 84 Lund, Sweden
[2] Lipid laboratory, Unit of Endocrinology, Department of Medicine, Karolinska Institute, Huddinge Stockholm, Sweden
Olga Göransson, Email: olga.goransson@med.lu.se
[*]Corresponding author

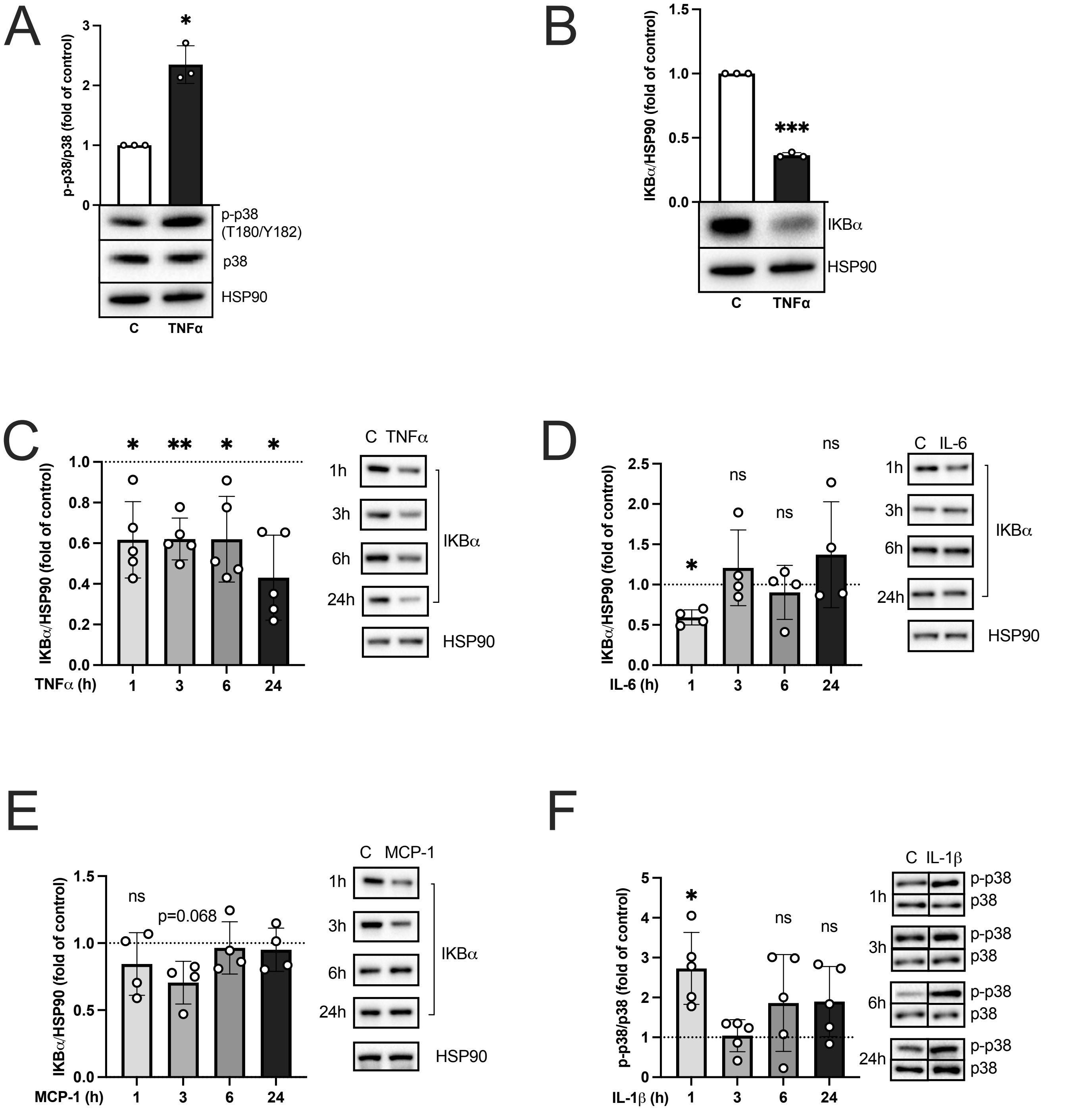

Supplementary Fig. S1:

p-p38 and IκBα protein levels at different timepoints after TNFα, IL-6, MCP-1 and IL-1β treatment

T180/Y185 p-p38/p38 ratio (A, F) and IκBα (B-E) protein levels in hMSCs differentiated into adipocytes (A-B) and 3T3-L1 adipocytes (C-F) treated with or without (control, C, dashed line) 50 ng/ml of human TNFα (A-B), 20 ng/ml of mouse TNFα (C), 20 ng/ml IL-6 (D), 20 ng/ml MCP-1 (E) or 20 ng/ml IL-1β (F) for 24h (A, B) or indicated time periods (C-F). HSP90 was used as a loading control. Representative blots are shown. Data are presented as means ± SD from multiple independent experiments (A-B; n=3, D-E; n=4, C, F; n=5). Statistical significance was analyzed by one sample t-test (A, B) or by one way ANOVA with a Holm-Šidak's multiple comparisons test (C-F). Uncropped blots are presented in Supplementary Figure S3 and Supplementary figure S4.

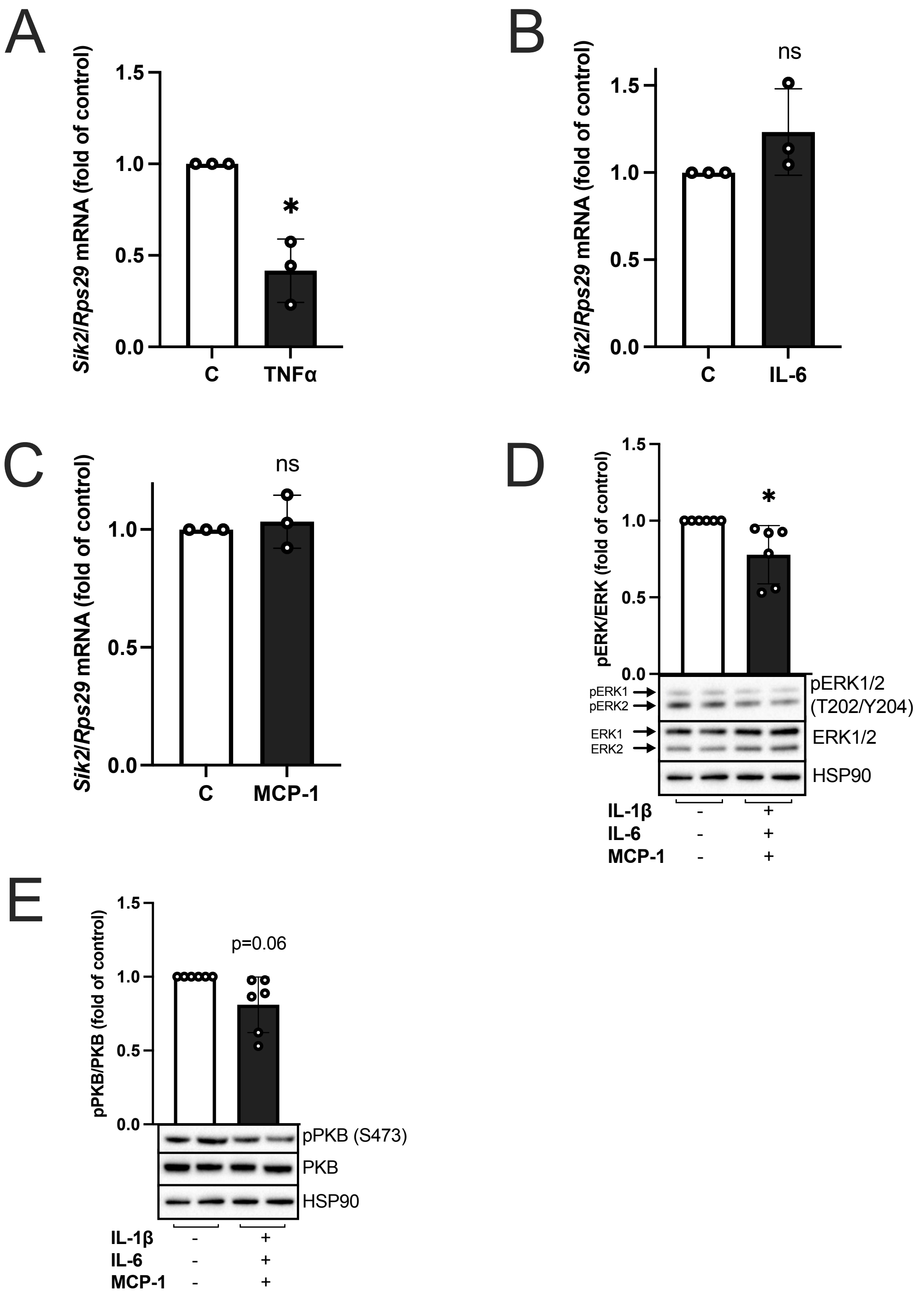

Supplementary Fig. S2:

Effect of TNFα, IL-6 and MCP-1 on *Sik2* mRNA levels and pERK/ERK and pPKB/PKB protein levels after treatment with a combination of IL-1β, IL-6 and MCP-1

Sik2 mRNA levels in 3T3-L1 adipocytes treated with 20 ng/ml TNFα (A), 20 ng/ ml IL-6 (B) and 20 ng/ml MCP-1 (C) for 24h. *Rps29* was used as a housekeeping gene for mRNA analysis. T202/Y204 pERK1/2/ERK1/2 ratio (D) and S473 pPKB/ PKB ratio (E) protein levels were assessed in 3T3-L1 adipocytes treated with a combination of 100 ng/ml IL-1β, 50 ng/ml IL-6 and 20 ng/ml MCP-1 for 24h (D-E). HSP90 was used as a loading control. Representative blots are shown. Data are presented as means ± SD from multiple independent experiments (A-C; n=3, D-E; n=6). Statistical significance was analyzed by one sample t-test. Uncropped blots are presented in Supplementary Figure S4.

Uncropped blots presented in Fig.1A

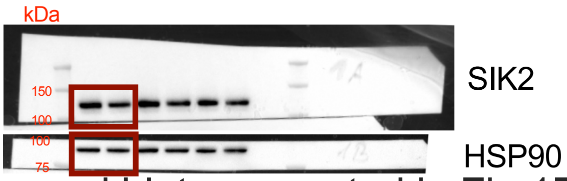

Uncropped blots presented in Fig.1B

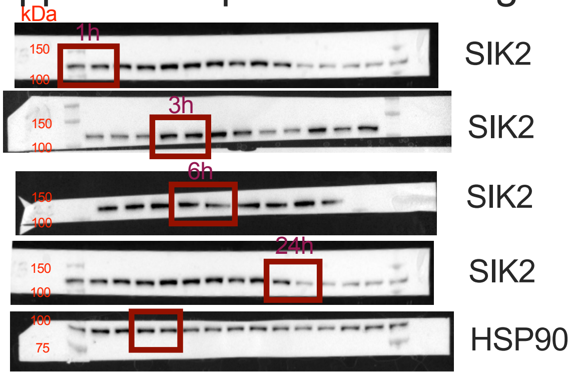

Uncropped blots presented in Fig.1C

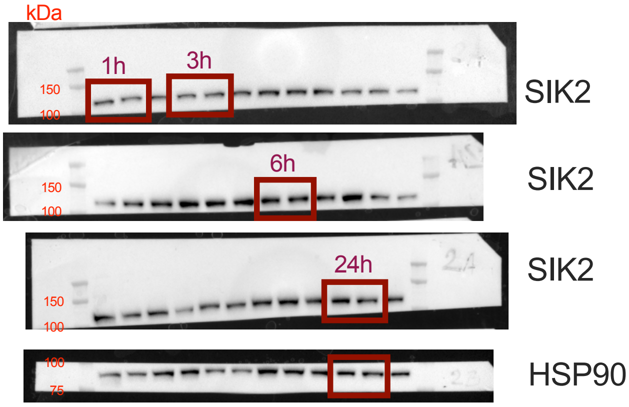

Uncropped blots presented in Fig.1D

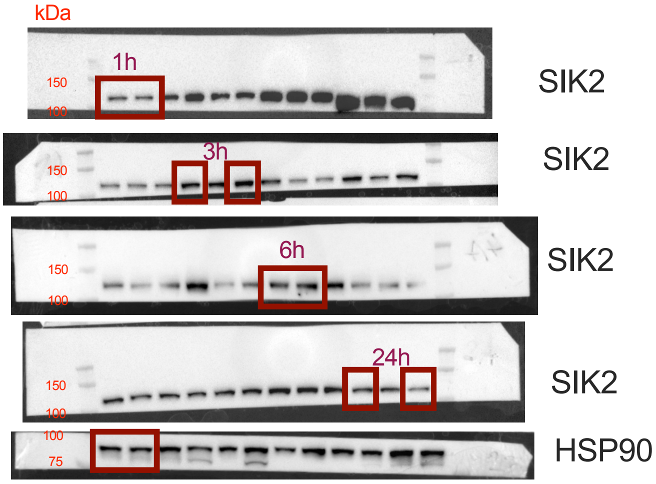

Uncropped blots presented in Fig.1E

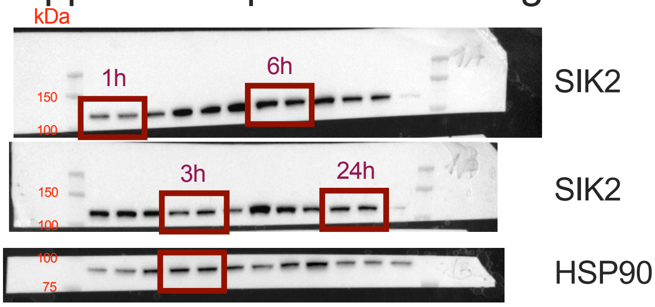

Uncropped blots presented in Fig.1G

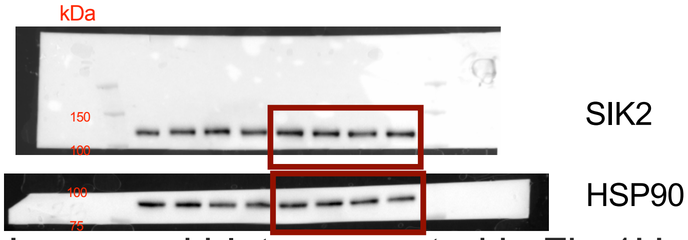

Uncropped blots presented in Fig.1H

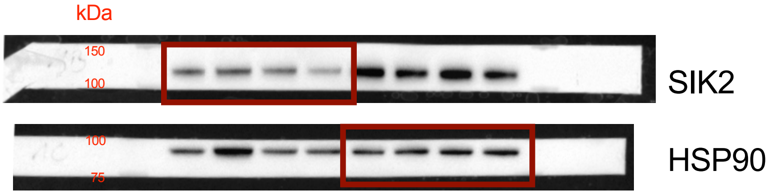

Uncropped blots presented in Fig.S1A

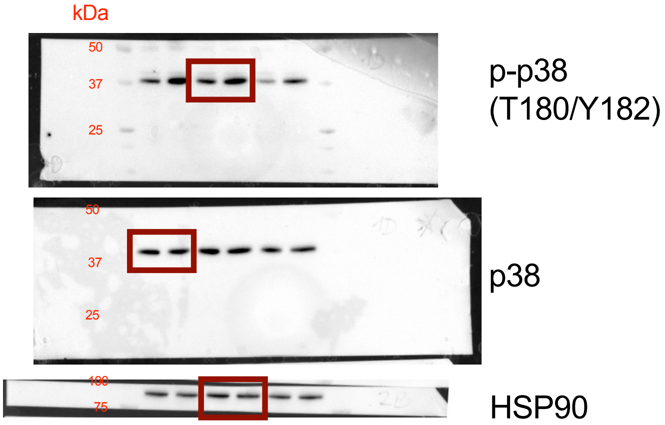

Uncropped blots presented in Fig.S1B

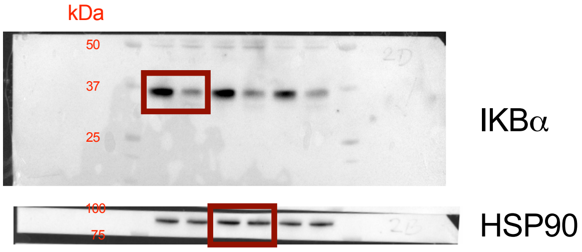

Uncropped blots presented in Fig.S1C

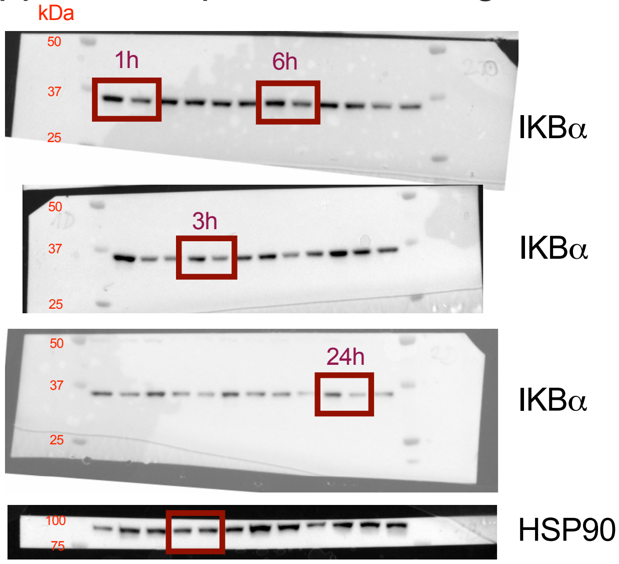

Supplementary Fig. S3:

Uncropped blots presented in Figure 1 and Supplementary figure S1A-C.

Bands marked with red square represent the bands shown in figures. Bands located between two red squares within one membrane in uncropped blots of Fig. 1C-D and Fig.S1C belongs to a cytokine-treated sample unrelated to the figure, which was loaded in the same gel for technical reasons.

Uncropped blots presented in Fig.S1D

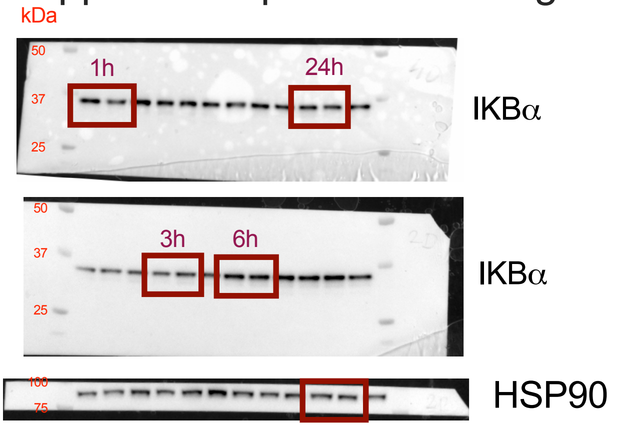

Uncropped blots presented in Fig.S1E

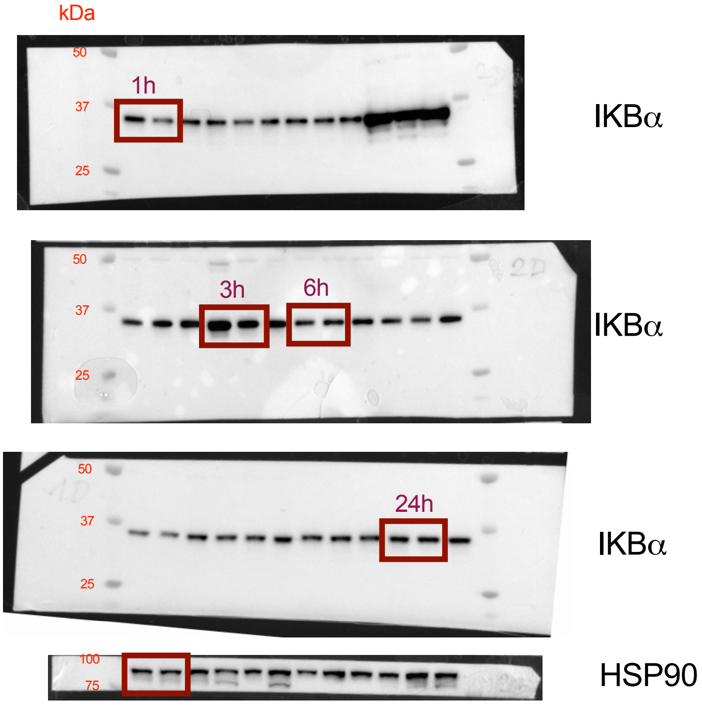

Uncropped blots presented in Fig.S1F

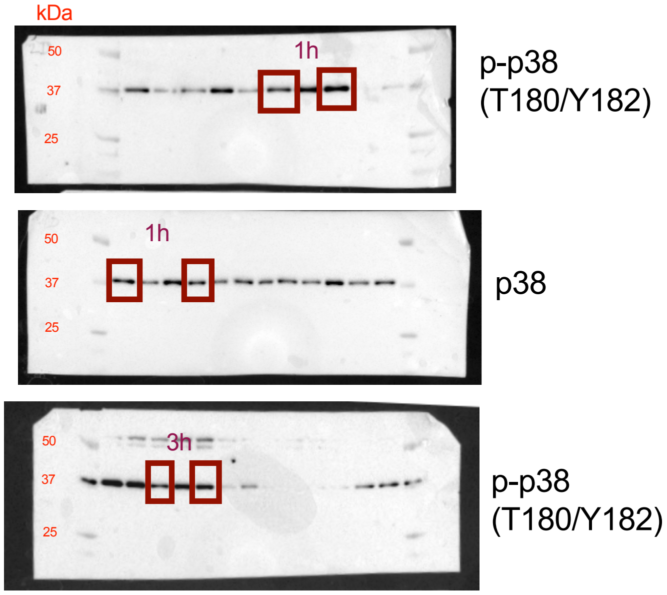

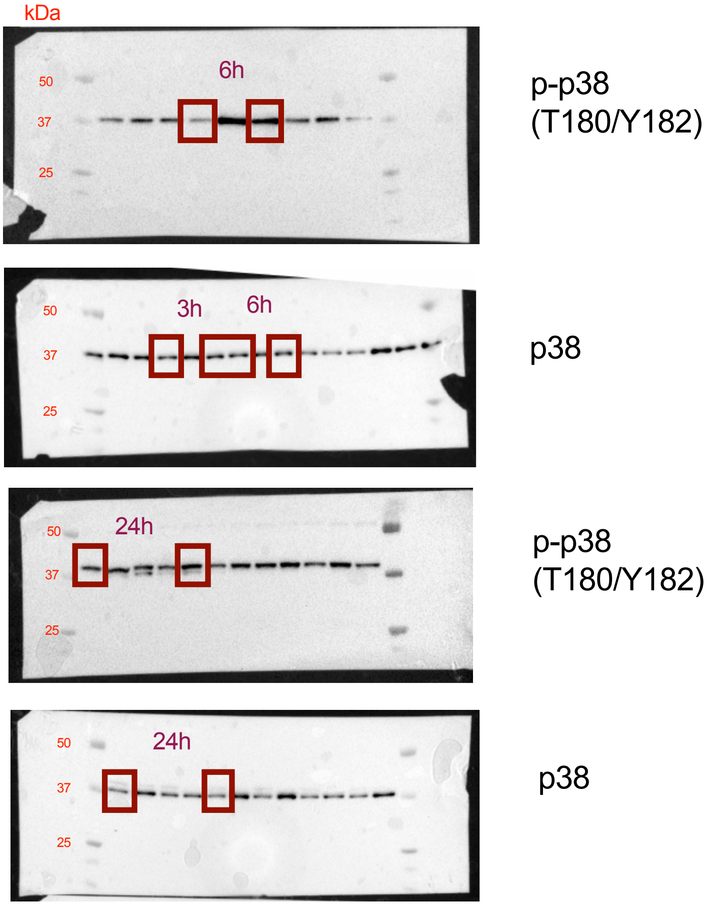

Uncropped blots presented in Fig.S2D

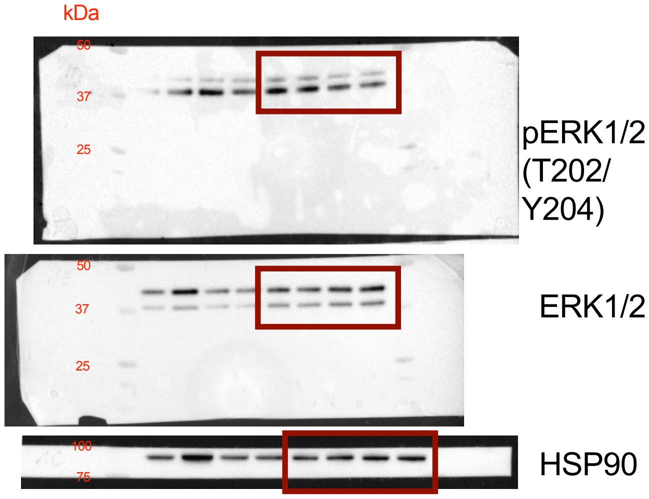

Uncropped blots presented in Fig.S2E

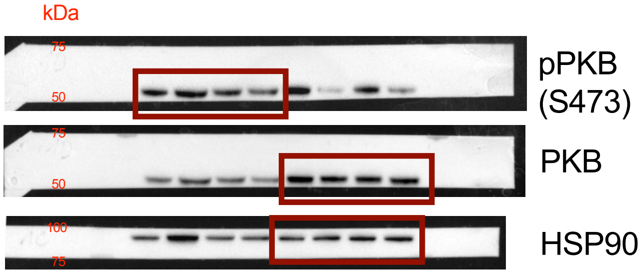

Supplementary Fig. S4: Uncropped blots shown in Supplementary figure S1D-F and in Supplementary figure S2.

Bands marked with red square represents the bands shown in figures. Bands located between two red squares within one membrane in uncropped blots of Fig. S1D-F belongs to cytokine unrelated to the figure and were loaded in the same gel for technical reasons.

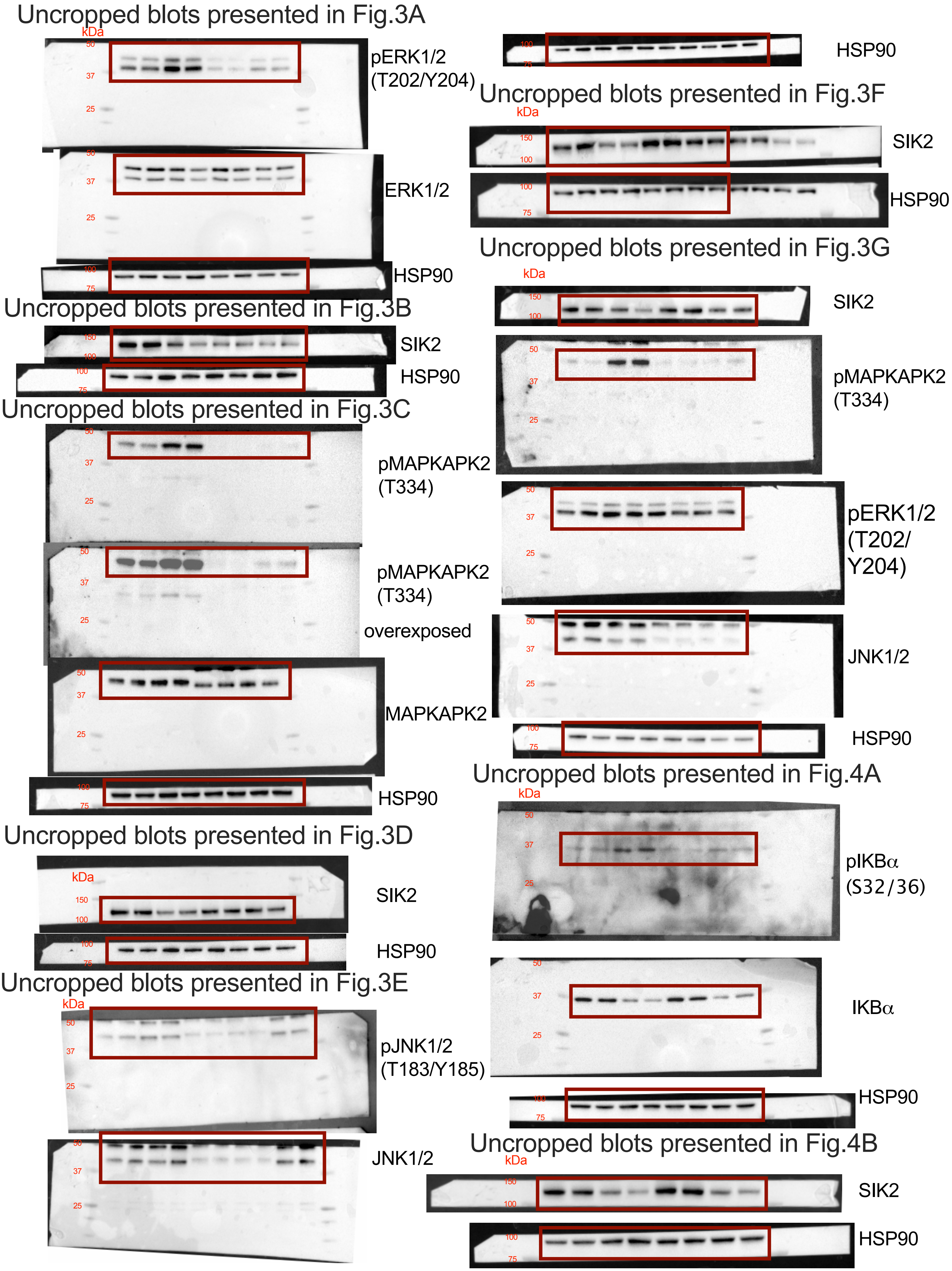

Supplementary Fig. S5: Uncropped blots shown in Figure 3 and Figure 4.

Bands marked with red square represent the bands shown in figures.
